# Biopsychological structure of Yin-Yang using Cloninger’s Temperament model and Carver and White’s BIS/BAS scale

**DOI:** 10.7717/peerj.2021

**Published:** 2016-05-18

**Authors:** Soo Jin Lee, Soo Hyun Park, Han Chae

**Affiliations:** 1 Department of Psychotherapy, Kyungil University, Daegu, South Korea; 2 Department of Psychology, Yonsei University, Seoul, South Korea; 3 School of Korean Medicine, Pusan National University, Busan, South Korea

**Keywords:** Yin Yang, Sasang personality questionnaire, BIS/BAS scale, Temperament and character inventory, Behavioral activation and inhibition, Emotionality

## Abstract

**Introduction**. The purpose of this study was to examine the psychological structure of Yin-Yang based on the Sasang Personality Questionnaire (SPQ) in relation to Carver and White’s Behavior Inhibition/Behavior Activation System (BIS/BAS) Scale and Cloninger’s temperament model of the West.

**Methods**. A total of 188 university students were classified as high (30%), middle (40%), and low (30%) groups based on their SPQ score and their differences in Cloninger’s temperaments and BIS/BAS subscales were analyzed using analysis of covariance after controlling the sex. Correlation among SPQ, Cloninger’s four temperaments and BIS/BAS subscales was also examined.

**Results**. Significant differences in BAS (*F* = 11.703, *p* < .001), Novelty-Seeking (*F* = 4.945, *p* < .01), and Harm-Avoidance (*F* = 10.912, *p* < .001) were observed between high and low SPQ score groups after controlling for sex. The SPQ showed significant correlation with BAS (*r* = 0.303), Novelty-Seeking (*r* = 0.225), and Harm-Avoidance (*r* = − 0.273). However, BIS showed no significant differences between SPQ groups, and did not show correlation with the SPQ.

**Discussion**. The current study demonstrated that Yin-Yang has similarities with and disparities from the Western tradition and may be examined with objective instruments. We showed that the emotionality of the East which is defined as mobility of emotion, not emotional instability as traditionally defined in Western theories, is pivotal for understanding the nature of emotion in the East. Suggestions are made for cross-cultural psychobiological study of the East and West.

## Introduction

Yin-Yang (*Eum* and *Yang* in Korean) is a representative term for describing two complementary and opposing traits of nature such as introvert–extrovert, female–male, passive–active, negative–positive, cold–hot, moon–sun, night–day, dark–bright, slow–fast, stable–dynamic, and so on (Heo, 1610; Lee et al., 2014b; Wu, Wu & Wang, 1997). Yin-Yang has been primary framework of philosophy, psychology, biology, sociology, medicine and technology for thousands of years in the East. However, Eastern traditional medicine is the only practical science based on Yin-Yang that has survived from the harsh modernization of the 19th century.

Nowadays, health care professionals across the world are increasingly paying attention to Eastern traditional medicine in order to overcome perceived limitations of Western biomedicine. Acupuncture and medical herbs are under the scope of many researchers in the West, and Eastern medicine has regained an equivalent status as Western medicine in Korea and China (Chae, 2015). Although acupuncture is regarded by some as a clinically effective medical technique, Yin-Yang as its basis is considered as a mere philosophical theory beyond the reach of scientific reasoning. The reason that Yin-Yang is regarded as forgotten wisdom may come from the fact that operational definition or acceptable objective tools necessary for scientific research had not been implemented yet.

There has been extensive investigation on the difference between the East and West and Yin-Yang as a foundation of such a difference from contextual, philosophical, religious, and cultural perspectives (Cheung, Van de Vijver & Leong, 2011; Clarke, 1994). For example, the cultural difference between collectivistic and individualistic societies were suggested as the major topic of East and West cross-cultural psychology (Cheung, Van de Vijver & Leong, 2011). However, Yin-Yang was merely described as the duality of the Eastern religion by Carl Jung and others (Clarke, 1994), and few studies have examined the biological aspects of Yin-Yang that has been fundamental for comprehending the biopsychosocial phenomena.

Therefore, proper considerations of Yin-Yang as a biopsychological principle would be needed for understanding human nature since the Eastern society has been treating its sick with their traditional psychology and psychiatry for thousands of years. For this purpose, we examined the biopsychological structure of Yin-Yang using Sasang Personality Questionnaire (SPQ) which measures the temperament perspectives of Yin-Yang based on Jema Lee’s Korean personalized medicine, Sasang typology, and has showed acceptable validity and clinical utility (Jang et al., 2012; Lee & Chae, 2014; Lee, Park & Chae, 2013c; Lee, Park & Chae, 2012; Lee et al., 2014b). The SPQ measures three domains of behavior, cognition and emotionality, and the *So-Yang* and *So-Eum* types representing Yang and Yin with high (30%) and low (30%) SPQ score were reported to have their typical psychological (Lee et al., 2014b), physical (Chae & Kown, 2015), pathophysiological (Lee et al., 2013a; Lee et al., 2013b), genetic (Kim et al., 2012; Sohn et al., 2012) and clinical (Lee et al., 2014b) characteristics (Table 1).

The Western temperament theory originated from four humors of Hippocrates and Galen, and it has been materialized with Extraversion and Neuroticism of Hans Eysenck which Jeffrey Gray rotated 45 degrees into the Behavioral Activation System (BAS) and the Behavioral Inhibition System (BIS) (Chae et al., 2014). Gray’s biological personality theory explains two fundamental motivational systems regulating approach and withdrawal behaviors to environmental stimuli (Li et al., 2014) which are responsible for affective states, personality and behavior, and their neuroanatomical and psychometric structures have been reported (Heym, Ferguson & Lawrence, 2008; Keiser & Ross, 2011; Torrubia et al., 2001). The BIS/BAS scale of Carver and White and Novelty-Seeking (NS) and Harm-Avoidance (HA) of Cloninger were reported to embody Gray’s temperament theory (Carver & White, 1994).

**Table 1:** General characteristics of So-Eum and So-Yang Sasang types.

Sasang Type (prevalence)	So-Eum (소음, 少陰) (30%)	So-Yang (소양, 少陽) (30%)
Origin of their nature	Enjoyment (樂) by wisdom (智)	Anger (怒) by righteousness (義)
	Worries can be relieved with wisdom. They enjoy what they have now.	They become angry when they are blocked. The anger can be regulated by fairness.
Temperament or Personality characteristics	Still, internally oriented, self-directed. Neat, mild, negative, intelligent, organized, patient, jealous, perseverant, passive, static, meticulous.	Active, externally oriented, talented for business. Unstable, easily get bored, sacrificing, righteous, easily acceptable, quick tempered, active, easy-going.
	Low SPQ. Low Novelty-Seeking and high Harm-Avoidance (TCI). Low Extraversion (NEO-PI). Low Positive Affect (PANAS) and high Trait Anxiety (STAI)	High SPQ. High Novelty-Seeking and low Harm-Avoidance (TCI). High Extraversion (NEO-PI).
Physical characteristics	Small Body Mass Index and Ponderal Index	Medium Body Mass Index and Ponderal Index with slightly developed muscles
Pathophysiological characteristics	Strong waste discharge, weak intake and digestion	Strong intake and digestion, weak waste discharge
Concerns for the maintaining good health	Good digestion. Maintain healthy digestive function, peristalsis, and body heat.	Easy with defecation. Avoid over-activation and overloads of bodily functions.
Frequent symptoms or diseases	Indigestion or dyspepsia, upper respiratory infection, neurotic symptoms	Constipation, gastroesophageal (laryngopharyngeal) reflux disease, affective disorder, insomnia, heat on chest
Type-specific medicinal herbs	Ginseng Radix, Atractylodis Rhizoma Alba, Glycyrrhizae Radix, Cinnamomi Cortex, Citri Pericarpium, Zingiberis Rhizoma Crudus	Rehmanniae Radix, Corni Fructus, Hoeoen, Alismatis Rhizoma, Osterici Radix, Angelicae Pubescentis Radix
Type-specific acupuncture points	Diagnose with HT7. Treatment with SP3(+)/ LI4(−)	Diagnose with HT3. Treatment with K13(+)/ SP3(−)

**Notes.**

TCI Temperament and Character Inventory NEO-PI NEO Personality Inventory SPQ Sasang Personality Questionnaire PANAS Positive and Negative Affect Schedule STAI State and Trait Anxiety Inventory

Previous studies presented similarities and disparities between Eastern Sasang typology and Western psychological theories including four humors of Hippocrates and Galen (Chae et al., 2009), Extraversion and Neuroticism (Chae et al., 2009), BIS/BAS scale (Lee et al., 2015) and NS and HA (Lee et al., 2014b; Park et al., 2011). The Choleric and Melancholic types of Galen have phenotypic similarities with So-Yang and So-Eum Sasang types, and the Extraversion and Neuroticism were supposed to be two major domains explaining Sasang typology (Chae et al., 2009). The Extraversion of Eysenck and McCrae were found to be useful explaining the psychological features of Sasang typology, however the Neuroticism was not as expected (Chae et al., 2009). As for the Gray’s temperament theory, the NS and HA successfully described So-Yang and So-Eum Sasang types (Lee et al., 2014b; Park et al., 2011), and the BIS/BAS scale was found useful (Lee et al., 2015).

**Figure 1:**
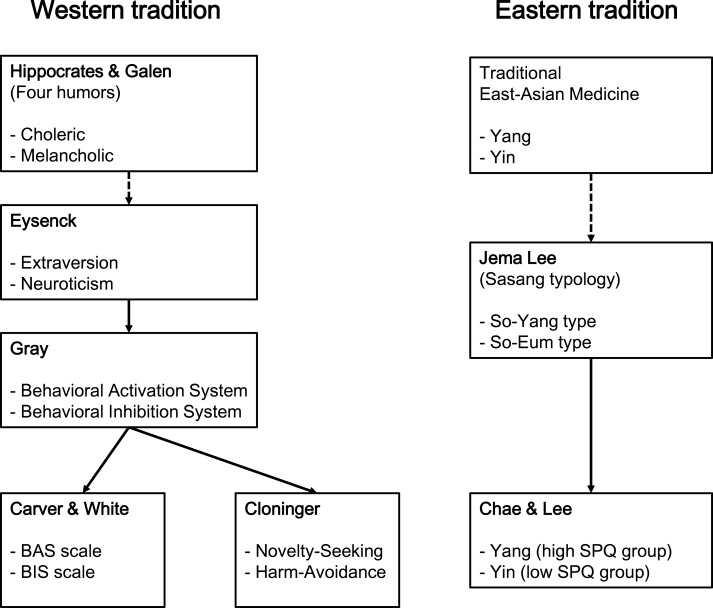
Biopsychology theories of West and East. Two opposite concepts or measures of each theory are paired.

In the present study, we analyzed the structure of Yin-Yang temperament using the SPQ concurrently with Carver and White’s BIS/BAS scale and Cloninger’s NS and HA to reveal potential differences in psychological structure (Fig. 1). The results in this study could contribute to establishing comprehensive understanding of human nature and provide foundation for integrative psychobiology across East and West.

## Methods

### Participants and measures

A total of 188 participants from 193 students of School of Korean Medicine who completed the Sasang Personality Questionnaire (SPQ) were included in this study and five participants who did not complete the SPQ were excluded from the study. In addition, Carver and White’s Behavioral Inhibition System/Behavioral Activation System (BIS/BAS) scale and Cloninger’s Temperament Character Inventory (TCI) were also measured. This study was approved by the Institutional Review Board of relevant institution (KMEDIRB2013-01). All participants provided written informed consent forms for the study.

#### Sasang Personality Questionnaire (SPQ)

The Sasang Personality Questionnaire (SPQ) is a 14-item self-report assessment developed for the objective assessment of psychological traits of Sasang typology which has theoretical basis on Yin-Yang and Confucianism. The SPQ is a sum of three subscales of personality components including SPQ-Behavior (SPQ-B) (e.g., passive vs. active), SPQ-Cognition (SPQ-C) (e.g., meticulous vs. easy-going), and SPQ-Emotionality (SPQ-E) (e.g., static vs. dynamic). Studies for its robust psychological structure and clinical validations have been reported, and the internal consistency of the SPQ, SPQ-B, SPQ-C, and SPQ-E was 0.81, 0.74, 0.62, and 0.62, respectively (Chae et al., 2014; Chae et al., 2012; Jang et al., 2012; Lee, Park & Chae, 2012; Lee, Park & Chae, 2013c).

The SPQ was found to be a valid instrument for differentiating Sasang types in adults, with the So-Yang repeatedly evidencing higher score than the So-Eum Sasang types (Chae et al., 2014; Jang et al., 2012; Lee, Park & Chae, 2012; Lee, Park & Chae, 2013c). In addition, it was reported that the high (30%) and low (30%) SPQ score group exhibited the biopsychological characteristics of So-Yang and So-Eum Sasang type groups as shown in Table 1 (Lee et al., 2015), which represent two opposite ends of temperament characteristics (Chae & Lee, 2015; Lee & Chae, 2014).

#### The Behavioral Inhibition System and Behavioral Activation System scale (BIS/BAS scale)

The BIS/BAS scale was developed by Carver and White for measuring the Behavioral Inhibition System (BIS, 7 items) and the Behavioral Activation System (BAS, 13 items); each item is scored using a 4 point Likert scale from ‘strongly disagree’ (1) to ‘strongly agree’ (4) (Carver & White, 1994). The robust psychometric structure was examined (Segarra et al., 2014; Smits & Boeck, 2006), and it was translated into Korean by Kim and Kim (Kim & Kim, 2001; Lee et al., 2015). The internal consistency of the BIS and BAS was 0.834 and 0.824, respectively.

BIS measures activation from aversive stimuli including punishment or non-reward which can be termed as anxiety, worry, and fear of making mistakes or social comparison. BAS is a sum of three subscales including BAS-Drive (BAS-D, 4 items), BAS-Fun Seeking (BAS-FS, 4 items), and BAS-Reward Responsiveness (BAS-RR, 5 items). The BAS-D measures the degree of pursuit of appetitive goals, BAS-FS reflects tendency to seek new potentially rewarding experiences and to act on the incentive of the moment, and the BAS-RR focuses on positive responses to the occurrence of reward (Carver & White, 1994; Segarra et al., 2014).

#### Temperament and Character Inventory (TCI)

Cloninger’s Temperament and Character Inventory (TCI) includes two-interrelated domains of temperament and character. Temperament traits reflect tendencies in automatic responses to emotional stimuli involving involuntary rational processes, whereas character traits depict differences in higher cognitive functions underlying a person’s goals, values, and relationships (Cloninger, 2008; Cloninger, Svrakic & Przybeck, 1993).

The temperament dimensions consist of Novelty Seeking (NS, characterized by exploratory excitability, impulsiveness, extravagance, and disorderliness), Harm Avoidance (HA, anticipatory worry, fear of uncertainty, shyness with strangers, and fatigability), Reward Dependence (RD, sentimentality, openness, attachment, and dependence), and Persistence (PS, eagerness, work-hardened, ambition, and perfectionism) (Chae et al., 2014). The Korean version of the Temperament and Character Inventory-Revised-Short (TCI-RS) (Min, Oh & Lee, 2007), a 140-item self-report questionnaire, asks individuals to rate each item on a 5-point scale (0 = not at all to 4 = very true). The internal consistency of NS, HA, RD and PS were reported as 0.829, 0.857, 0.814, and 0.821, respectively.

### Statistical analysis

The demographic features of gender, education, and age in high, middle, and low SPQ score groups were described. The high, middle, and low SPQ score groups were assigned based on the order of SPQ score of each subject. The higher 30% and lower 30% were calculated first, and the rest were allocated as the middle SPQ score group. *χ*^2^ test for gender and education level and analysis of variance (ANOVA) for age were used for the differences between high, middle, and low SPQ score groups.

Analysis of Covariance (ANCOVA) was used to examine the significant differences between high, middle, and low SPQ score groups in BIS/BAS and TCI temperament subscales after controlling for sex, and the post-hoc analysis with Bonferroni was used. Pearson’s correlation was used among SPQ, BIS/BAS, and TCI temperament subscales and the correlation coefficients were acquired.

Statistical results were presented as frequency (%) and mean ± standard deviation and statistical significance level was set at *p* < 0.05. IBM SPSS Statistics 20.0 (IBM, Armonk, NY) was used for all statistical analyses.

## Results

### Demographic characteristics of participants

188 participants (90 males and 98 females; 21–63 years, mean age: 29.80; mean education years: 16.58 years) were divided into three groups based on SPQ scores. The SPQ score of the low SPQ score group (*n* = 56, 29.8%) was lower than 24, that for the high SPQ score group (*n* = 56, 29.8%) was higher than 30, and that of the middle SPQ group (*n* = 76, 40.4%) was between 25 and 29 (Table 2). No significant differences in gender, age and educational level were observed among high, middle, and low SPQ groups.

**Table 2:** Demographic features of high (30%), middle and low (30%) SPQ score groups. Biopsychological structure of Yin-Yang using Cloninger’s Temperament model and Carver and White’s BIS/BAS scale. The high, middle and low SPQ score group was allocated based on the percentile order of SPQ score.

	High SPQ (30≤)	Middle SPQ (25≤ and ≤29)	Low SPQ (≤24)	
Male/female	22/34	38/38	30/26	*χ*^2^ = 2.521, *df* = 2, *p* = .283
Age	29.37 ± 4.26	29.26 ± 4.31	30.94 ± 6.54	*F* = (2, 185) = 2.056, *p* = 0.131
Education				*χ*^2^ = .907, *df* = 2, *p* = .635
Bachelor	38	57	39	
Master	18	19	17	

**Notes.**

SPQ Sasang Personality Questionnaire

### Differences in SPQ, BIS/BAS, and TCI temperament subscales between high and low SPQ score groups

Significant differences in SPQ, BIS/BAS, and TCI temperament subscales after controlling for sex except on the BIS were observed between high and low SPQ score groups (Table 3). In the post-hoc analysis of ANCOVA, Bonferroni was used for the analysis of significant differences between groups.

**Table 3:** SPQ, TCI temperament and BIS/BAS subscales in high, middle and low SPQ score groups.

	High SPQ	Middle SPQ	Low SPQ		Post-hoc in ANCOVA
SPQ^***^	33.64 ± 2.88	26.78 ± 1.30	21.14 ± 2.35	*F* = 457.642, *p* < .001	high > middle > low
SPQ-B^***^	12.34 ± 1.98	10.22 ± 1.90	7.11 ± 1.32	*F* = 121.620, *p* < .001	high > middle > low
SPQ-C^***^	11.79 ± 1.66	9.55 ± 1.53	7.70 ± 1.85	*F* = 81.901, *p* < .001	high > middle > low
SPQ-E^***^	9.52 ± 1.60	7.00 ± 1.80	6.34 ± 1.83	*F* = 50.228, *p* < .001	high > low, middle
HA^***^	34.48 ± 11.39	36.25 ± 11.15	43.67 ± 11.89	*F* = 10.912, *p* < .001	high, middle > low,
NS^**^	35.13 ± 10.82	33.03 ± 9.00	29.52 ± 9.70	*F* = 4.945, *p* < .01	high > low
RD^*^	48.25 ± 8.45	46.97 ± 8.18	43.60 ± 9.56	*F* = 3.754, *p* < .05	high > low
PS^**^	50.64 ± 9.92	47.87 ± 9.03	43.91 ± 10.05	*F* = 7.283, *p* < .01	high > low
BIS	20.25 ± 3.31	19.61 ± 4.04	20.68 ± 3.48	*F* = 1.570, *p* > .05	
BAS^***^	39.65 ± 5.43	38.47 ± 4.55	35.29 ± 4.60	*F* = 11.703, *p* < .001	high, middle > low
BAS-D^**^	11.33 ± 2.25	10.79 ± 1.71	9.91 ± 2.03	*F* = 7.069, *p* < .01	high, middle > low
BAS-FS^***^	11.96 ± 2.21	11.57 ± 2.09	10.09 ± 2.14	*F* = 11.978, *p* < .001	high, middle > low
BAS-RR^*^	16.37 ± 2.02	16.11 ± 2.30	15.29 ± 2.09	*F* = 3.383, *p* < .05	

**Notes.**

* *p* < .05

** *p* < .01

*** *p* < .001

SPQ Sasang Personality Questionnaire TCI Temperament and Character Inventory BIS/BAS Behavioral Inhibition System and Behavioral Activation System scale SPQ-B SPQ-Behavior SPQ-C SPQ-Cognition SPQ-E SPQ-Emotionality HA Harm-Avoidance NS Novelty-Seeking RD Reward-Dependence PS Persistence BIS Behavior Inhibition System BAS Behavior Activation System BAS-D BAS-Drive BAS-FS BAS-Fun Seeking BAS-RR BAS-Reward Responsiveness

The estimated means and standard errors of the high SPQ score group was significantly higher than that of the low SPQ score group on SPQ total score (33.57 ± .29 and 21.19 ± .29), SPQ-B (12.33 ± .24 and 7.11 ± .24), SPQ-C (11.76 ± .22 and 7.72 ± .22), and SPQ-E (9.48 ± .23 and 6.36 ± .23), respectively. The estimated means and standard errors of the high SPQ score group showed significantly higher score than those of the low SPQ score group in NS (35.26 ± 1.31 and 29.44 ± 1.32), RD (48.03 ± 1.16 and 43.75 ± 1.17), and PS (50.79 ± 1.29 and 43.81 ± 1.30), respectively. In contrast, the estimated mean and standard error of the high SPQ score group showed lower HA (34.25 ± 1.53 and 43.83 ± 1.54) than that of the low SPQ score group.

The estimated means and standard errors of the high SPQ score group showed significantly higher score than those of the low SPQ score group in BAS (39.62 ± .38 and 35.31 ± .55), BAS-D (11.33 ± .27 and 9.91 ± .27), BAS-FS (11.97 ± .30 and 10.08 ± .29)and BAS-RR (16.32 ± .30 and 15.32 ± .29). However no significant differences (*F* = 1.570, *p* = .211) in the estimated mean and standard errors of BIS (20.11 ± .50 and 20.77 ± .48) were observed between high and low SPQ score group.

### Correlation among SPQ, BIS/BAS, and TCI temperament subscales

The correlation coefficients between BIS/BAS scale and TCI temperament subscales are shown in Table 4. The BIS was positively correlated with HA (*r* = .593, *p* < .001) and negatively with NS (*r* = − .220, *p* < .01), the BAS was negatively correlated with HA (*r* = − .176, *p* < .05) and positively with NS (*r* = .621, *p* < .001) suggesting the possibility that the concept of Yang is similar to that of BAS and NS as well as the concept of Yin is similar to that of BIS and HA.

**Table 4:** Correlation coefficients between subscales of TCI temperament and BIS/BAS scale.

	HA	NS	RD	PS
BIS	.593^***^	−.220^**^	.128	−.149^*^
BAS	−.176^*^	.621^***^	.232^**^	.371^***^
BAS-D	−.161^*^	.426^***^	.167^*^	.445^***^
BAS-FS	−.301^***^	.707^***^	.077	.201^**^
BAS-RR	.052	.316^***^	.298^***^	.238^**^

**Notes.**

* *p* < .05

** *p* < .01

*** *p* < .001

Bold represents coefficients larger than 0.3.

TCI Temperament and Character Inventory HA Harm-Avoidance NS Novelty-Seeking RD Reward-Dependence PS Persistence BIS Behavior Inhibition System BAS Behavior Activation System BAS-D BAS-Drive BAS-FS BAS-Fun Seeking BAS-RR BAS-Reward Responsiveness

However, the correlation coefficients between SPQ and BIS/BAS and TCI temperament subscales presented in Table 5 demonstrate the different conceptualization of Yin and Yang between the West and East. The SPQ showed significantly positive correlation with BAS (*r* = .303, $\underline{p}\lt .001$), NS (*r* = .225, *p* < .01) and negative correlation with HA (*r* = − 237, *p* < 001), but not with BIS (*r* = − .024, *p* > .05). While the SPQ was positively correlated with BAS and NS corresponding to the concept of Yang, the SPQ was significantly correlated only with HA, suggesting that the concept of BIS is a somewhat different idea of Yin from the perspective of the West.

**Table 5:** Correlation coefficients between subscales of SPQ and TCI temperament and BIS/BAS scale.

	TCI temperament				BIS/BAS scale				
	HA	NS	RD	PS	BIS	BAS	BAS-D	BAS-FS	BAS-RR
SPQ	−.273^***^	.225^**^	.237^**^	.255^***^	−.024	.303^***^	.261^***^	.279^***^	.176^*^
SPQ-B	−.353^***^	.207^**^	.325^***^	.313^***^	−.010	.284^***^	.288^***^	.207^**^	.182^*^
SPQ-C	−.274^***^	.170^*^	.005	.199^**^	−.143	.254^**^	.234^***^	.269^***^	.098
SPQ-E	.054	.120	.179^*^	.031	.102	.121	.034	.144	.102

**Notes.**

* *p* < .05

** *p* < .01

*** *p* < .001

Bold represents coefficients larger than 0.3.

SPQ Sasang Personality Questionnaire TCI Temperament and Character Inventory BIS/BAS Behavioral Inhibition System and Behavioral Activation System scale SPQ-B SPQ-Behavior SPQ-C SPQ-Cognition SPQ-E SPQ-Emotionality HA Harm-Avoidance NS Novelty-Seeking RD Reward-Dependence PS Persistence BIS Behavior Inhibition System BAS Behavior Activation System BAS-D BAS-Drive BAS-FS BAS-Fun Seeking BAS-RR BAS-Reward Responsiveness

## Discussion

This study analyzed the biopsychological structure of Yin-Yang, a central dogma of traditional Eastern science, using Carver and White’s BIS/BAS scale and Cloninger’s HA and NS temperament dimensions which have been affected by Gray’s biopsychological theory. The biopsychological perspective of Yin-Yang is needed to understand Eastern psychology and psychiatry (Heo, 1610; Lee et al., 2014b; Wu, Wu & Wang, 1997), and yet it was not implemented as a major topic until now.

**Figure 2:**
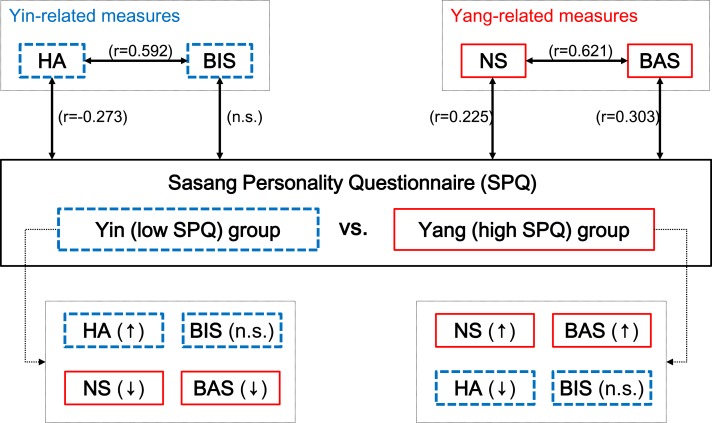
Illustrated relation among Carver and White’s BIS/BAS scale, Cloninger’s NS and HA, and Chae and Lee’s SPQ in this study. Yin and Yang groups are two opposite ends of Sasang Personality Questionnaire; Yang is a high (30%) SPQ score group who is active, easy-going and dynamic, while, Yin is a low (30%) SPQ score group who is passive, meticulous and static. Yang-related measures are marked with red solid line, and Yin-related measures are marked with blue dotted line.

Yin-Yang has two kinds of attributes: Since Yin and Yang are two complementary and independent factors as well as two opposite ends of a single dimension at the same time, we analyzed correlations between temperament scales of BIS/BAS, NS/HA and SPQ, and differences between high and low SPQ score groups representing Yang and Yin concurrently. In the present study, the SPQ showed positive correlation with Cloninger’s NS and Carver’s BAS. The NS and BAS score of the high SPQ score group were significantly higher than that of the low SPQ score group after controlling for sex (Fig. 2). NS is a heritable tendency in the activation or initiation of behaviors such as frequent exploratory activity in response to novelty, impulsive decision making, and extravagance in approach to cues of reward (Chae et al., 2014). BAS was known to be crucial for approach behavior and reward-related motivation (Li et al., 2014). Considering that BAS is highly correlated with NS, these two have significant similarities in its conceptualization. To summarize, Yang can be defined as an inclination for behavioral activation, impulsiveness, approach to novel things, response to rewards, and concern for outside goals.

The SPQ showed negative correlation with Cloninger’s HA, but not with Carver’s BIS. The HA score was significantly lower in the high SPQ score group than in the low SPQ score group, but the BIS was not (Fig. 2). The associations between SPQ and HA and between BIS and HA were as expected, but the relationship between SPQ and BIS were not as expected. HA, which is negatively correlated with SPQ, is an inherited tendency of inhibition or cessation of behaviors as a result of anticipatory worries, passive avoidant behaviors from uncertainty and shyness of strangers (Chae et al., 2014; Park et al., 2011). Furthermore, BIS is reported to be correlated with response to fear-related stimuli (Mathews, Yiend & Lawrence, 2004), which also plays an important role in the modulation of anxiety-related behaviors, worries and negative emotions (Li et al., 2014). This suggests that BIS is distinctive on the discontinuation or termination of behavior from fear and anxiety, while HA emphasizes an active behavioral modification to an opposite direction (Carver & White, 1994; Segarra et al., 2014). From these correlations among SPQ, HA and BIS, we might define Yin as active discontinuation or modification of behaviors to opposite direction to avoid harm without apparent emotional instability.

The recognition of emotionality or emotional instability would be a critical biopsychological factor in explaining the disparities between the East and West. SPQ-emotionality, unlike other subscales of the SPQ (SPQ-behavior and SPQ-cognition), did not significantly correlate with Carver and White’s BIS/BAS scale and Cloninger’s NS and HA in this study (Table 5). Previously (Lee, Park & Chae, 2012), the SPQ showed no significant correlation (*r* = − 0.168, n.s.) with Neuroticism of NEO-Personality Inventory measuring level of emotional instability, anxiety and negative emotionality (Carver & White, 1994; Smits & Boeck, 2006). This implies that the emotionality of the East is defined as a level of emotional mobility (static vs. dynamic) which has neutral meaning compare to the emotional instability (fear or anxiety) of the Western theories. In addition, the East has regarded emotional instability (fear or neurotic) as a state to be controlled with education and learning of Confucianism which deems human as a proactive existence characterized by self-awareness and propensity towards self-directed character development (Lee et al., 2014a; Lee et al., 2014b). This kind of traditional Eastern wisdom would give us multidimensional and insightful understandings on the human nature (Chae, 2015).

The findings of this study could be summarized into the embodiment of an operational definition of Yin-Yang from the perspective of temperament in traditional Eastern medicine. Yang would be defined as an approach to novel stimuli which can be intensified by rewards, and Yin would be an active shift to an opposite direction without emotional instability (Chae et al., 2009; Lee, Park & Chae, 2013c; Lee et al., 2014b; Park et al., 2011). Although this should be tested in further studies, it may be the distinctive biopsychological characteristics of traditional Eastern medicine when compared to the Western.

Yin-Yang has been an essential theory for the traditional Eastern medicine, and has been used for the prevention, diagnosis, treatment and prevention of human disease. All of the classic books of Eastern medicine have recognized Yin and Yang as two major super factors of the psychological temperament. For example, the *Yellow Emperor’s Canon of Internal Medicine* (circa 722 B.C.), which is considered as the bible of the Eastern medicine, described that joy and anger are emotional representatives of Yin and Yang (Wu, Wu & Wang, 1997) based on the mobility of the emotion. The *Principle of Life Preservation in Eastern Medicine* (1894) of Sasang typology defined the enjoyment and gladness as Yin, and anger and sorrow as Yang (Lee, 1894). Also, the Sasang typology rephrased Yin and Yang in moral and ethical perspectives in which everybody dislikes the bad (wrong) and likes the good (right) (Lee, 1894). These notes of Yin-Yang from Eastern psychology happens to be similar to Freud’s pleasure-pain principle that the mind seeks pleasure and avoids pain to satisfy biopsychological needs (Freud, 1952). As shown in this study, the contrasting and complementary biological concepts of the West, such as Behavioral Inhibition and Activation, Anxiety and Impulsivity, Harm-avoidant and Novelty-approaching is also similar to that of Yin and Yang.

There still lies limitation for generalization of the results. This study suggested an operational definition for Yin-Yang in biopsychology and raised the emotionality as a factor explaining the difference between the Eastern and Western psychological tradition for the first time, and it should be replicated again using objective instruments with consideration of age, sex, education, and cultural and ethnic background. In addition, the SPQ should be tested with neuroanatomical and psychometric structures (Chae et al., 2014; Chae et al., 2009) which was suggested as dopamine projections associated with NS and BAS and serotonin projections associated with HA and BIS (Bray, Shimojo & O’Doherty, 2010; Heck et al., 2009; Li et al., 2014; Munafo et al., 2003; Munafo et al., 2008; Simon et al., 2010).

This study examined the psychological structure of SPQ with BIS/BAS scale and Cloninger’s HA and NS (Figs. 1 and 2) and reviewed its biopsychological meanings in respect to Yin-Yang theory, which has been the fundamental basis of traditional Eastern medicine for thousands of years. The interpretation of emotionality is different between the East and West, such that emotional mobility is more applicable in the East while emotional instability is more characteristic in the West. It provides a foundation for understanding the Eastern psychological theories and integration of Eastern and Western traditions with further studies.

## Supplemental Information

10.7717/peerj.2021/supp-1 Supplemental Information 1 Raw data

## Additional Information and Declarations

### Data Availability

The following information was supplied regarding data availability: Raw data is available as Supplemental Information.
